# The Cause of Cancer

**Published:** 1901-02-23

**Authors:** 


### THE CAUSE OF CANCER.

THE discussion as to the cause of cancer continues to swing from side to side. No one now seems to doubt the fact that in many forms of cancer certain suspicious looking bodies are found which have been by some regarded as the cancer parasite. The nature of these bodies has been much discussed; many of the theories put forward from time to time with the object of explaining them without admitting their parasitic nature, have been shown to be untenable; and thus a steady drift of opinion towards the acceptance of the parasitic theory has lately been observable. Now, however, we find strong arguments brought forward to show that these bodies are but red blood cells in various stages of degeneration. It has been shown by Olt that the reaction of the cancer bodies in sections is similar in many respects to that of the red blood corpuscles, and that like them also they contain iron. After all, however, the question does not seem to be one that can properly be decided by any amount of histological or chemical similarity. It is to cultivation and inoculation experiments that we must turn if the matter is to be satisfactorily answered, and in this direction the difficulties are great. The problem has been largely widened by recent researches in other fields. It is becoming abundantly clear that in many diseases which are truly parasitic direct infection does not take place, and direct experimental inoculation must of necessity fail. Thus, while we rightly ask for demonstration of the transmissibility of cancer before we accept its parasitic nature as proven, we cannot admit that failure to inoculate is any disproof of its being a parasitic disease. The byways of infection are far too wide and far too mysterious, and the whole question is at present far too much in a state of ferment for us to allow for a moment that any amount of experimental failure can disprove such a theory. All they can do is to show that so far we have failed to hit upon the particular medium through which the infection is transmitted, or to discover the details of the life-cycle through which the parasite must pass for its full development. It is a big question; too big a one for the microscope or the test tube to decide.

